# Shrub Invasion Overrides the Effect of Imposed Drought on the Photosynthetic Capacity and Physiological Responses of Mediterranean Cork Oak Trees

**DOI:** 10.3390/plants12081636

**Published:** 2023-04-13

**Authors:** Raquel Lobo-do-Vale, Teresa Rafael, Simon Haberstroh, Christiane Werner, Maria Conceição Caldeira

**Affiliations:** 1 Forest Research Centre, Associate Laboratory TERRA, School of Agriculture, University of Lisbon, 1349-017 Lisbon, Portugal; 2 Ecosystem Physiology, Faculty of Environment and Natural Resources, University of Freiburg, 79110 Freiburg, Germany

**Keywords:** photosynthesis, *V_cmax_*, *J_max_*, water stress, climate change, shrub encroachment, *Quercus suber*, *Cistus ladanifer*

## Abstract

Mediterranean ecosystems face threats from both climate change and shrub invasion. As shrub cover increases, competition for water intensifies, exacerbating the negative effects of drought on ecosystem functioning. However, research into the combined effects of drought and shrub invasion on tree carbon assimilation has been limited. We used a Mediterranean cork oak (*Quercus suber*) woodland to investigate the effects of drought and shrub invasion by gum rockrose (*Cistus ladanifer*) on cork oak carbon assimilation and photosynthetic capacity. We established a factorial experiment of imposed drought (ambient and rain exclusion) and shrub invasion (invaded and non-invaded) and measured leaf water potential, stomatal conductance and photosynthesis as well as photosynthetic capacity in cork oak and gum rockrose over one year. We observed distinct detrimental effects of gum rockrose shrub invasion on the physiological responses of cork oak trees throughout the study period. Despite the imposed drought, the impact of shrub invasion was more pronounced, resulting in significant photosynthetic capacity reduction of 57% during summer. Stomatal and non-stomatal limitations were observed under moderate drought in both species. Our findings provide significant knowledge on the impact of gum rockrose invasion on the functioning of cork oak and can be used to improve the representation of photosynthesis in terrestrial biosphere models.

## 1. Introduction

Shrub invasion is a growing concern globally [1], including in Mediterranean ecosystems [2], potentially impacting ecosystem functioning [3]. Mediterranean ecosystems are seasonally exposed to drought stress [4], due to dry and hot summers. The invasion of shrubs in these areas is likely to worsen soil drying [2,5], and intensify the effects of drought stress [6]. While these ecosystems have evolved to tolerate the drought stress periods during the summer (e.g., [4,7]), the forecasted increase in the frequency and intensity of droughts [8], concomitantly with the invasion of shrubs, might jeopardize the functioning of these ecosystems.

Mediterranean cork oak (*Quercus suber*) woodlands are particularly interesting model systems to study the interaction between shrub invasion and drought. These ecosystems are being increasingly invaded by the shrub gum rockrose (*Cistus ladanifer*) [3,9,10] which has been linked to cork oak tree mortality and failure of natural regeneration [10,11,12]. These oak woodlands are vital silvopastoral ecosystems in the western Mediterranean region, providing significant ecosystem services and economic value mostly due to cork production (for more details see, e.g., [3,13]). Cork oak is an evergreen tree, well adapted to the Mediterranean climate, and highly resilient to drought [14], while gum rockrose is a native, semi-deciduous shrub, highly competitive for water [6]. In previous studies, it has been shown that gum rockrose shrub invasion negatively affected tree water balance by increased competition for water [2,5,6,15], that even resulted in lower cork oak tree growth [15,16].

Plant species that have a conservative water use, such as cork oak, limit water losses at the onset of drought by closing their stomata [17,18], but this strategy comes at a cost of decreasing the amount of carbon assimilated [14,19], which can negatively impact their growth and other processes [14,20]. Conversely, plant species such as gum rockrose, that are much less water conservative [6,21], and maintain more open stomata during drought [22], experience significant water losses but are able to fix more carbon [21,23,24]. It was also shown that the hydraulic behavior of both species was altered from the dry to the wet season and that gum rockrose shrub invasion significantly impacted the hydraulic strategy of cork oak trees during drought conditions [5]. However, apart from causing higher stomatal limitation, drought stress can negatively affect photosynthetic capacity through effects on the maximum activity of Rubisco carboxylation and/or electron transport [4,25,26], for example, by changes in the activity of enzymes such as Rubisco. Indeed, hydraulic limitations can also result in reduced nutrient uptake and transport, affecting the plant’s metabolic activity and further hindering growth. Nevertheless, there is a lack of knowledge on the seasonality of photosynthetic capacity of both species and on how shrub invasion can combine with drought to alter cork oak photosynthetic capacity. Understanding the interplay between hydraulic limitations and photosynthetic capacity is critical for predicting plant responses to water stress and managing the impacts of water scarcity on these ecosystems. This knowledge not only provides an in-depth analysis of plant physiology but is also needed for integration into large-scale vegetation models, to better simulate long-term photosynthesis and reduce modelling uncertainties under future climate change scenarios. Similarly, a better understanding of the susceptibility of Mediterranean tree species, such as cork oak, to shrub invasion and their biotic interactions, can provide valuable insights into the underlying mechanisms driving these interactions and help anticipate the impacts of shrub invasion on ecosystem functioning and develop adaptative management strategies that promote the resilience and sustainability of these ecosystems, particularly in conditions of increasing aridity [8,27].

The main objective of this study was to investigate the combined effects of imposed drought and shrub invasion by the gum rockrose on the physiological behavior and photosynthetic capacity of cork oak trees, evaluated by the apparent maximum rate of Rubisco carboxylation (*V_cmax_*) and apparent maximum rate of electron transport (*J_max_*). To achieve our aims, we established a shrub invasion and imposed drought field experiment on a cork oak woodland invaded by gum rockrose shrubs. Tree and shrub water status, leaf carbon assimilation and photosynthetic capacity were monitored over one year, in ambient and imposed drought conditions.

## 2. Results

### 2.1. Meteorological Conditions

**Figure 1:**
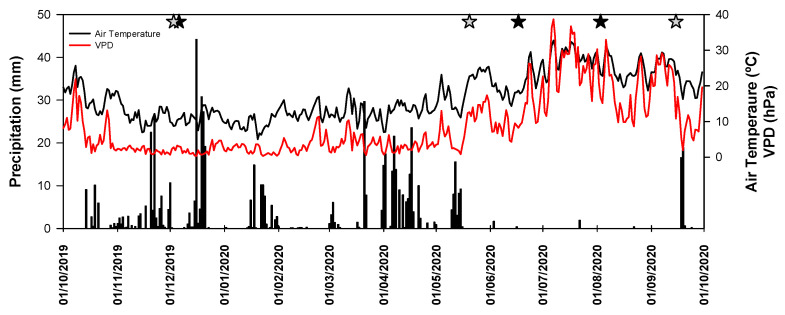
Precipitation (mm), air temperature (°C) and VPD (hPa) during the hydrological year of 2019/2020. The black stars indicate the sampling days of leaf gas exchange and leaf water potential, and grey stars indicate the sampling days of *A/Ci* response curve measurements.

The hydrological year of 2019/2020 was characterized by a mean air temperature of 16.9 °C and a total rainfall of 660 mm (Figure 1). The imposed drought treatments reduced the total amount of precipitation to only 363 mm. The highest mean temperature of the year was observed in July (28.2 °C) and the lowest in January (9.3 °C). In comparison to the long-term mean (1981–2010, Évora, [28]), no significant overall deviations were observed (16.9 °C versus 16.5 °C), apart from a heat wave that occurred in July during summer (+4.3 °C). The total precipitation was 13% higher in comparison with the long-term average. The sampling periods (indicated by the grey and black stars in Figure 1) in December 2019 and May 2020 were preceded by significant precipitation events. The field campaigns performed from June to September monitored the onset and progression of summer water stress (Figure 1).

### 2.2. Effects of Imposed Drought and Shrub Encroachment on Cork Oak Physiological Responses

**Figure 2:**
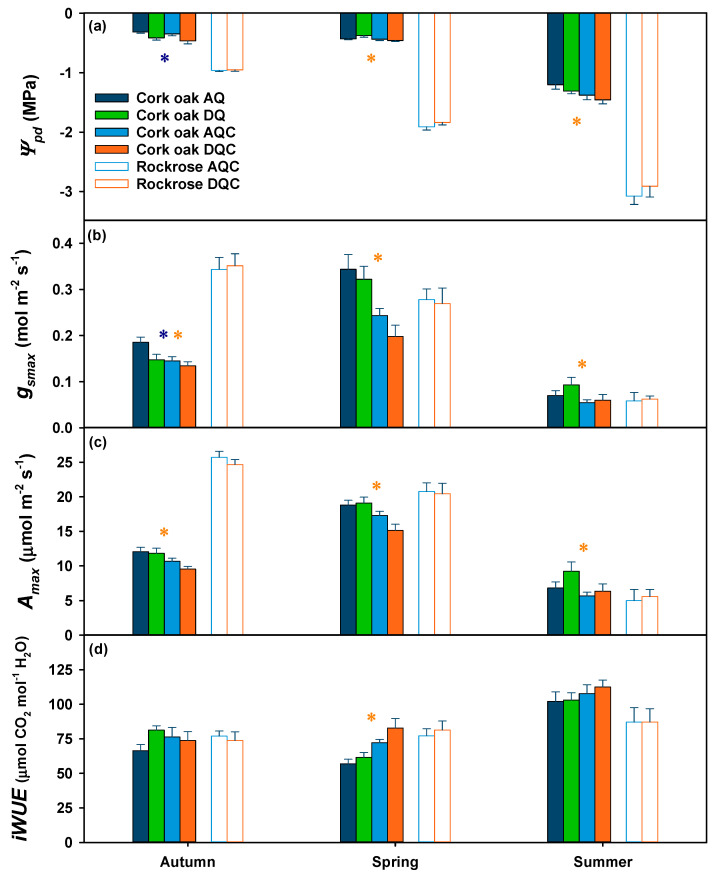
Predawn leaf water potential (*Ψ_pd_*) (**a**), stomatal conductance at which maximal carbon assimilation was observed (*g_smax_*) (**b**), maximal carbon assimilation (*A_max_*) (**c**) and intrinsic water use efficiency (*iWUE*) (**d**) in cork oak trees (filled bars) subjected to the four treatments (AQ, AQC, DQ, DQC) and gum rockrose shrubs (open bars) subjected to drought treatment (AQC and DQC). The bars represent the mean ± standard error. The blue asterisk denotes a significant effect of imposed drought, and the orange asterisk denotes a significant effect of invasion within each date (*p* < 0.05).

Predawn leaf water potential (*Ψ_pd_*), stomatal conductance (*g_smax_*) and maximal photosynthetic rate (*A_max_*) of cork oak trees (Figure 2, filled bars) varied significantly between dates (*p* < 0.001) and were predominantly affected by the gum rockrose shrub invasion (*p* = 0.003 for *Ψ_pd_*, and *p* < 0.001 for *g_smax_* and *A_max_*), while the overall effect of imposed drought was only observed in autumn *Ψ_pd_* (*p* = 0.025).

High values of predawn leaf water potential (*Ψ_pd_*, Figure 2a, filled bars) indicated that cork oak trees were fully hydrated in autumn and spring, but progressed to moderate water stress in summer. The mean values (±standard error) ranged from −0.38 ± 0.02 MPa in autumn to −1.34 ± 0.04 MPa in summer. During autumn, trees subjected to drought treatments (DQ—imposed drought, non-invaded by shrubs; DQC—imposed drought, invaded by shrubs) had significantly (*p* = 0.006) lower *Ψ_pd_* compared to those in the ambient treatments (AQ—ambient precipitation, non-invaded by shrubs; AQC—ambient precipitation, invaded by shrubs), despite good water status in all treatments. In the following dates, the invasion of shrubs surpassed the effect of drought, resulting in significantly (*p* = 0.047 for spring and *p* = 0.021 for summer) lower *Ψ_pd_* in trees from invaded treatments (−1.38 ± 0.08 and −1.46 ± 0.07 MPa in AQC and DQC treatments, respectively) when compared to non-invaded treatments (−1.20 ± 0.07 and −1.31 ± 0.04 MPa in AQ and DQ treatments, respectively).

Leaf gas exchange measurements, specifically *g_smax_* and *A_max_*, showed an increase from autumn to spring and then a sharp decrease during summer (Figure 2b,c, filled bars). In autumn, even though the trees had high *Ψ_pd_*, their leaf gas exchange values were intermediate when compared to spring or summer. Trees subjected to imposed drought (DQ and DQC) showed significantly (*p* = 0.027) lower *g_smax_* (−16%) compared to trees in ambient treatments (AQ and AQC) in autumn. Additionally, trees in invaded treatments (AQC and DQC) had significantly lower *g_smax_* than those in non-invaded treatments (AQ and DQ) on all dates (*p* = 0.015, *p* < 0.001 and *p* = 0.049, for autumn, spring and summer, respectively). The highest values of leaf gas exchange were observed in the spring in non-invaded trees, and these values were significantly higher than those observed in trees in invaded treatments (*p* = 0.001 for *A_max_*). In summer, the invasion by shrubs led to a more pronounced limitation in leaf gas exchange of trees, resulting in a greater reduction in *g_smax_* (−74%) compared to *A_max_* (−63%, *p* = 0.050).

In spring, the trees in non-invaded and shrub-invaded treatments had *g_smax_* values of 0.33 ± 0.02 mol m^−2^ s^−1^ and 0.22 ± 0.02 mol m^−2^ s^−1^, respectively, and *A_max_* values of 19.0 ± 0.5 μmol m^−2^ s^−1^ and 16.2 ± 0.6 μmol m^−2^ s^−1^, respectively. In summer, *g_smax_* values decreased to 0.08 ± 0.01 mol m^−2^ s^−1^ and 0.06 ± 0.0 mol m^−2^ s^−1^, and *A_max_* values to 8.0 ± 0.8 μmol m^−2^ s^−1^ and 6.0 ± 0.6 μmol m^−2^ s^−1^, in trees in non-invaded and shrub-invaded treatments, respectively.

The pattern of intrinsic water use efficiency (*iWUE*, Figure 2d, filled bars) reflected the observed higher stomatal closure (*g_smax_*) compared to carbon assimilation (*A_max_*) variations. *iWUE* was overall higher (*p* = 0.007) in trees in invaded treatments although just significant in the spring (*p* < 0.001). The trees showed the highest *iWUE* in summer, with values of 102.6 ± 4.1 μmol CO_2_ mol^−1^ H_2_O for non-invaded trees and 110.1 ± 4.0 μmol CO_2_ mol^−1^ H_2_O for invaded trees.

### 2.3. Effects of Imposed Drought on Gum Rockrose Physiological Responses

Throughout the study period, the physiological responses of gum rockrose shrubs (*Ψ_pd_*, *A_max_* and *g_smax_*, open bars in Figure 2) were not significantly affected by the imposed drought (*p* > 0.05). Instead, they were primarily determined by the ambient conditions (*p* < 0.001 for date effect on *Ψ_pd_*, *A_max_* and *g_smax_*). The *Ψ_pd_* of gum rockrose shrubs decreased from autumn (−0.96 ± 0.01 MPa) to summer (−2.99 ± 0.11 MPa), leading to a progressive reduction in *g_smax_*, which in turn limited *A_max_*. In summer, the shrubs exhibited a strong stomatal closure, with a *g_smax_* of 0.06 ± 0.01 mol m^−2^ s^−1^, in contrast to the 0.35 ± 0.02 mol m^−2^ s^−1^ values recorded during autumn. This closure of stomata severely inhibited photosynthesis (*A_max_*), with a photosynthetic rate of 5.30 ± 0.92 μmol m^−2^ s^−1^ in summer, which was notably lower than the 25.18 ± 0.58 μmol m^−2^ s^−1^ photosynthetic rate recorded in autumn. No significant changes over time or effects of imposed drought (*p* > 0.05) were noted in *iWUE* (Figure 2d, open bars). The *iWUE* showed a slight non-significant increase from autumn to summer in both treatments (75.3 ± 3.6 and 87.1 ± 6.9 μmol CO_2_ mol^−1^ H_2_O in autumn and summer, respectively).

### 2.4. Photosynthetic Capacity (V_cmax_ and J_max_) of Cork Oak Trees and Gum Rockrose Shrubs

The photosynthetic capacity of both species was overall not significantly affected by either drought or shrub invasion (*p* > 0.05) but varied with the prevailing environmental conditions (*p* < 0.001 for date effect). However, when the statistical analysis was performed by date and species, a significant effect of gum rockrose shrub invasion was observed in summer in *V_cmax_* and *J_max_* (*p* = 0.047 and *p* = 0.049, respectively).

**Figure 3:**
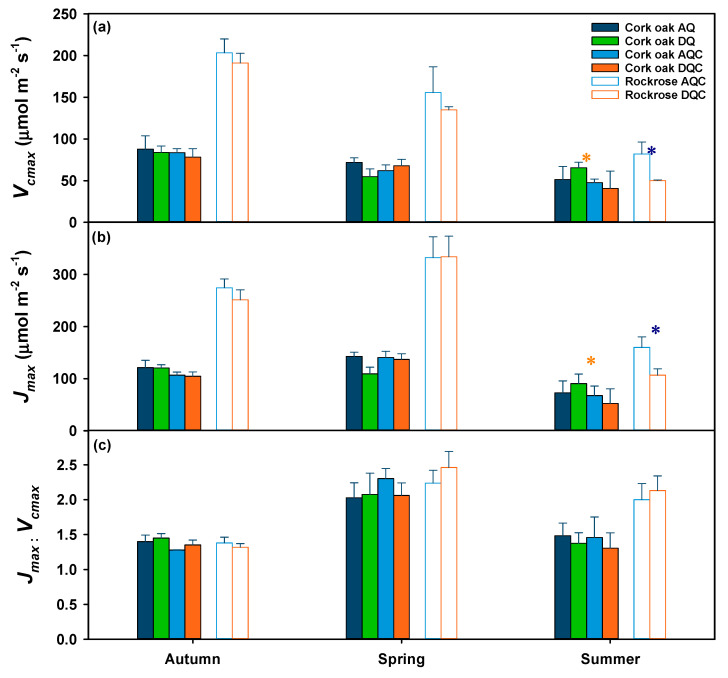
Maximum Rubisco carboxylation rate (*V_cmax_*) (**a**), and maximum electron transport rate (*J_max_*) (**b**) in cork oak trees (filled bars) subjected to the four treatments (AQ, AQC, DQ, DQC) and gum rockrose shrubs (open bars) subjected to drought treatment (AQC and DQC)(**c**). The bars represent the mean ± standard error. The blue asterisk denotes a significant effect of drought, and the orange asterisk denotes a significant effect of invasion within each date (*p* < 0.05).

The maximum rate of Rubisco carboxylation (*V_cmax_*) in cork oak trees decreased significantly from autumn to summer (Figure 3a, filled bars). *V_cmax_* values were highest in autumn (83.3 ± 4.2 μmol m^−2^ s^−1^) and lowest in summer (50.4 ± 15.3 μmol m^−2^ s^−1^). The maximum electron transport rate (*J_max_*, Figure 3b, filled bars) had no significant differences between autumn and spring but decreased significantly in summer. The highest *J_max_* values (134.0 ± 5.9 μmol m^−2^ s^−1^) were observed in spring and the lowest (69.6 ± 24.1 μmol m^−2^ s^−1^) in summer. As a result of differing patterns of *V_cmax_* and *J_max_*, the *J_max_*/*V_cmax_* varied significantly (*p* < 0.001) between 1.4 ± 0.2 (autumn and summer) and 2.1 ± 0.1 (spring), with no significant effects of imposed drought or shrub invasion.

The *V_cmax_* of the gum rockrose shrub decreased steadily and significantly (*p* < 0.001) from autumn to summer (Figure 3a, open bars). The highest *V_cmax_* overall mean was observed in autumn (198.1 ± 10.5 μmol m^−2^ s^−1^), while the lowest was in summer (65.9 ± 9.65 μmol m^−2^ s^−1^). Still, the imposed drought had a significant negative effect (*p* = 0.040) on *V_cmax_* of shrubs during summer, with treatment means of 81.9 ± 14.5 μmol m^−2^ s^−1^ and 50.0 ± 0.9 μmol m^−2^ s^−1^, in the AQC and DQC treatments, respectively. The *J_max_* (Figure 3b, open bars) increased from autumn to spring, but decreased in summer. The highest *J_max_* values (333.0 ± 26.2 μmol m^−2^ s^−1^) were observed in spring, while summer had the lowest overall mean (133.3 ± 15.9 μmol m^−2^ s^−1^). As for *V_cmax_*, the imposed drought significantly (*p* < 0.001) decreased *J_max_* of the shrubs in summer (159.9 ± 20.3 μmol m^−2^ s^−1^ and 106.7 ± 12.2 μmol m^−2^ s^−1^ in AQC and DQC treatments, respectively). *J_max_*/*V_cmax_* ranged from 1.4 ± 0.1 (autumn) to 2.3 ± 0.1 (spring).

**Figure 4:**
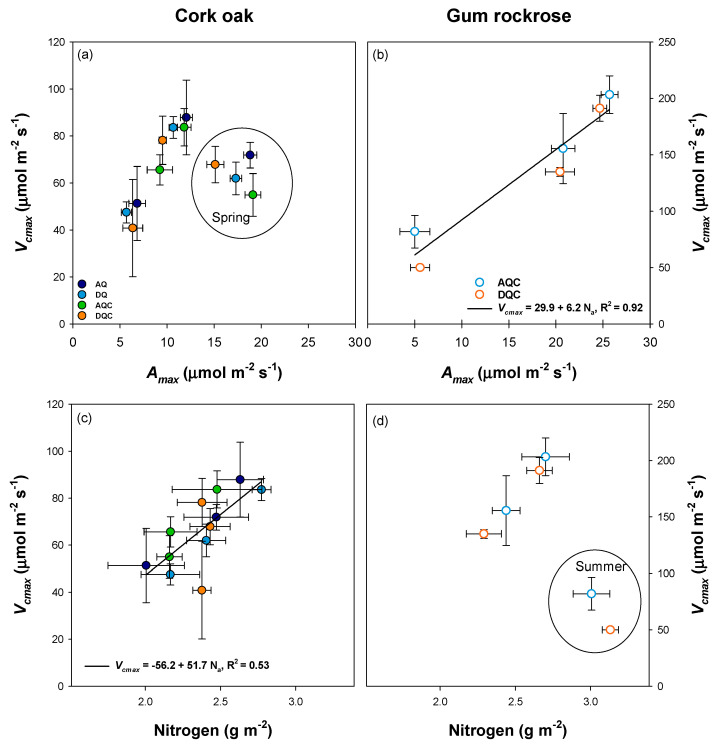
Relationships between maximum Rubisco carboxylation rate (*V_cmax_*) and maximal photosynthetic rate (*A_max_*) in cork oak (**a**) and gum rockrose (**b**). Relationships between maximum Rubisco carboxylation rate (*V_cmax_*) and leaf nitrogen content in an area basis (Nitrogen) in cork oak (**c**) and gum rockrose (**d**). The bars represent the mean ± standard error for each treatment in each season. The circles isolate the spring data in (**a**) and the summer data in (**d**). Note the different scales for cork oak and gum rockrose on the relationships presented.

There was no overall significant correlation between *V_cmax_* and *A_max_* in cork oak trees (*p* = 0.379, Figure 4a), possibly due to the low spring *V_cmax_*. However, *V_cmax_* was positively and significantly correlated with leaf nitrogen content (*N_a_*, *p* = 0.007, Figure 4c). In gum rockrose, a significant correlation was observed between *V_cmax_* and *A_max_* (*p* = 0.002, Figure 4b), but not between *V_cmax_* and *N_a_* (*p* = 0.188, Figure 4d).

## 3. Discussion

Our study demonstrates a clear negative impact of the gum rockrose shrub invasion on the physiological functioning of cork oak trees across the three seasons we investigated. Interestingly, the effect of shrub invasion was found to override the impact of the drought treatment, ultimately leading to an impairment of the photosynthetic capacity during the summer. Moreover, we did not observe an overall significant effect of imposed drought on the maximum Rubisco carboxylation rate (*V_cmax_*) and maximum electron transport rate (*J_max_*) of cork oak trees, but only a significant effect of shrub invasion on *V_cmax_* and *J_max_* during summer.

Shrub invasion negatively affected cork oak tree photosynthetic responses, most probably due to increased competition for water. In fact, the negative effects of shrub invasion on tree water balance were recently reported, particularly during drought events [5,6,15]. The gum rockrose shrub has high photosynthetic rates and an anisohydric water-spending strategy [5,24,29,30,31]. This competitive water usage led to a decrease in the *Ψ_pd_*, *g_smax_* and *A_max_* of cork oak trees in invaded treatments. Cork oak is a drought-avoiding species, which has a conservative water use, with a strong stomatal control [14,18,32]. By minimizing water loss through stomatal control and extracting water from deep soil reserves [33,34], cork oak is able to maintain carbon assimilation, resulting in higher *iWUE*. The greater impact of shrub invasion than imposed drought on tree functioning highlights the significant role of shrub competition for water resources and supports the findings from previous studies [5,15] which suggested that the water demand of invasive shrubs, such as gum rockrose, can surpass the impact of drought stress on the ecosystem [6]. Indeed, the minor overall effect of imposed drought on cork oak and gum rockrose physiological responses may be attributed to the frequency and amount of precipitation of the study year (660 mm), where heavy rain events were interspersed with dry spells, smoothing the differences between ambient and imposed drought treatments. Haberstroh et al. (2021) [15] also observed that the effects of imposed drought were more pronounced in periods with sufficient water supply, in agreement with our observations of a significant effect of drought in *Ψ_pd_* only in autumn, when the trees had an overall good water status. Nevertheless, the cork oak trees displayed some level of stomatal inhibition and hence a reduction in carbon assimilation in the autumn when compared to spring, when the highest values were observed (Figure 2b,c). Conversely, gum rockrose recorded the highest values of *g_smax_* and *A_max_* during autumn. These differences are probably related to differences in the optimal temperature for photosynthetic metabolism of each species [35] and the high plasticity of gum rockrose to adapt to different conditions [6].

The photosynthetic capacity variation was not consistent between species or biochemical parameters (*V_cmax_* and *J_max_*), although both species displayed a significant decline in both parameters during the summer (Figure 3). Furthermore, while the effects of shrub invasion were significant only in summer for cork oak trees, imposed drought effects were significant also only in summer for gum rockrose. The values of *V_cmax_* and *J_max_* observed in our study are in agreement with previous studies reported for cork oak with contrasting water availabilities [36,37] or in studies with other *Quercus* or sclerophylls [26,38,39]. Similarly, the observed values of *V_cmax_* and *J_max_* for gum rockrose in our study were like those reported for gum rockrose during the summer in the only study that we are aware of [40], but also for other sclerophyllous shrubs [26,39,41].

In cork oak, *V_cmax_* peaked in autumn (83.3 ± 4.2 μmol m^−2^ s^−1^), significantly decreasing in spring to 64.8 ± 3.6 μmol m^−2^ s^−1^ (Figure 3a), when *A_max_* was highest (Figure 2c). This resulted in no significant relationship between *V_cmax_* and *A_max_* (Figure 4a). However, *V_cmax_* showed a significant relation with leaf nitrogen concentration per unit area (*N_a_*, Figure 4c). These results suggest a down-regulation of *V_cmax_* in spring in cork oak according to the least-cost theory [42,43], which posits that the costs of carboxylation and water loss during photosynthesis are balanced with the costs of acquisition and maintenance of resources (e.g., nitrogen) needed for photosynthesis. By allocating less nitrogen to the photosynthetic metabolism, or Rubisco in particular, while maintaining high *A_max_*, more nitrogen can be allocated to other sinks, such as the growth of new leaves or other tree components. Considering that major cork oak growth occurs during spring [20], this strategy will allow the trees to maximize nitrogen use for growth. This can be regarded as a nutrient conservation strategy, as nitrogen can be used for growth or stored for later use, when less nitrogen is available from the soil, for example, during drought. In fact, this is confirmed by the observation that nitrogen remobilization efficiency in cork oak trees was higher during drought years [20]. *N_a_* was recently regarded as the consequence and not the cause of *V_cmax_* [44], due to the higher environmental controls on *V_cmax_* [45]. These results highlight that the allocation of nitrogen is a more important determinant of photosynthetic capacity than total amount of nitrogen [45]. This seems to be the case in cork oak, attending to the lack of correlation of *N_a_* with *A_max_*.

On the other hand, the *V_cmax_* of gum rockrose peaked in autumn and steadily decreased until summer (Figure 3a), showing a linear relationship with *A_max_* (Figure 4b). However, *V_cmax_* was not correlated with *N_a_*, (Figure 4d), due to the high leaf nitrogen concentrations in summer when *A_max_* was impaired (Figure 2c). Gum rockrose is a semi-deciduous shrub that responds to drought by leaf abscission during the summer, as stress becomes more severe [23]. The high leaf nitrogen concentration might be attributed to the translocation of nitrogen from the senescent to the active leaves, while the photosynthetic apparatus is impaired. This strategy may enable gum rockrose to recover quickly after the first autumn rains. Due to its shallow root system and the maintenance of photosynthetically active leaves during the dry period, gum rockrose can respond promptly to precipitation events after the summer [6,21].

A strong relationship between *V_cmax_* and *J_max_* was initially proposed by Wullschleger [39], but recent developments suggest a trade-off between *J_max_* relative to *V_cmax_* [46], as well as a remarkable seasonal variability in the two photosynthetic parameters [47]. Our results are in agreement with these findings, as *J_max_*/*V_cmax_* varied over time and between species. While the range of variation was similar between cork oak and gum rockrose (roughly 1.4 to 2.3, Figure 3c), the differences between species in summer were noticeable, with a lower ratio in cork oak compared to gum rockrose. These differences highlight once again the different water-use strategies of the species. The conservative behavior of cork oak is also manifested in the lower *J_max_*/*V_cmax_*, indicating a coordination of *J_max_* to *V_cmax_* to avoid photoinhibition, by lowering the electron transport capacity when Rubisco carboxylation activity is limited [46].

At the leaf level, it is well established that stomata strongly limit the rate of CO_2_ assimilation under water stress conditions [19,48]. While non-stomatal limitations can also occur under prolonged drought conditions [26,49,50], the biochemical limitations are not as relevant until the drought becomes severe [17,26,51,52]. Our results show that even moderate water stress during the summer can impair photosynthesis in shrubs and trees, with both strong stomatal limitations (lower Ci and lower *g_smax_*, Figure 2) and non-stomatal limitations (lower *V_cmax_* and *J_max_*, Figure 3).

Comparison of *V_cmax_* and *J_max_* between the two species indicated that gum rockrose had a much higher photosynthetic capacity than cork oak trees, during autumn and spring. Our findings are consistent with the traits of gum rockrose shrub, such as their dense and shallow root system and anisohydric behaviour, which enables it to respond quickly to precipitation and maintain high stomatal conductance and photosynthetic rates. These adaptations give gum rockrose shrubs a competitive advantage over cork oak trees for water resource use.

However, in summer, the inhibition of the photosynthetic capacity was overall higher in gum rockrose, with reductions of 67% and 60% of maximum *V_cmax_* and *J_max_*, respectively. This inhibition was further exacerbated by the imposed drought in gum rockrose, with reductions of 70% for both *V_cmax_* and *J_max_*. In cork oak trees, overall reductions in photosynthetic capacity were also noticeable during summer, with reductions of 40% and 39% of maximum *V_cmax_* and *J_max_*, respectively, and they were exacerbated by invasion of gum rockrose, with reductions of 45% and 56% for *V_cmax_* and *J_max_*, respectively. Stomatal limitations (*g_smax_*) were also more severe in gum rockrose shrubs, with reductions of 82% compared to 75% in cork oak trees in relation to maximum observed values. As such, in both species in addition to stomatal limitations, non-stomatal limitations of photosynthesis have also occurred, as demonstrated by the lower values of *V_cmax_* and *J_max_*. However, mesophyll conductance was also observed to decrease under drought conditions, potentially contributing to the reduction in apparent *V_cmax_* [4,26,37]. Despite cork oak showing high mesophyll conductance values (around 0.1 mol CO_2_ m^−2^ s^−1^) compared to other evergreen oaks [26,37,53], the mesophyll conductance in gum rockrose [40] and other malacophylls is about double (> 0.2 mol CO_2_ m^−2^ s^−1^ [26,37,41]. The contribution of mesophyll limitations for the whole non-stomatal limitations in each species remains to be clarified.

In conclusion, the results of this study indicate that gum rockrose shrub is a highly competitive species that has evolved effective adaptations to maintain high levels of photosynthetic assimilation and water use even under mild water stress. Its dense and shallow root system allows it to respond quickly to precipitation and maintain high stomatal conductance and photosynthetic rates, giving it a competitive advantage over other species like cork oak. The gum rockrose shrub follows a “spender strategy”, where it invests resources to maximize productivity while environmental conditions allow it, even at a cost of a steep decrease in functioning during drier summer conditions. In contrast, cork oak follows a “maintenance strategy”, closing stomata earlier at the cost of carbon assimilation. While this strategy ensures less variability in tree functioning, it will negatively affect tree growth. Indeed, a previous study conducted at the site revealed significantly lower growth in invaded trees compared to non-invaded ones, as evidenced by LAI and trunk diameter measurements [15]. This highlights the urgent need to develop adaptive management strategies that foster resilience and sustainability of Mediterranean ecosystems, especially in the face of increasing aridity [8,27]. Failure to do so may result in shrub invasion triggering drastic changes in ecosystem structure and function [54]. The study also found that non-stomatal (biochemical) limitations of photosynthesis occur in both species under moderate water stress, as demonstrated by the lower values of *V_cmax_* and *J_max_*.

Overall, these findings provide important insights into the effect of gum rockrose invasion on cork oak functioning and on the ecology of these species and their responses to changing environmental conditions, which can also be used to improve the terrestrial biosphere models’ accuracy in predicting ecosystem responses to climate and land-use changes.

## 4. Materials and Methods

### 4.1. Study Site and Experimental Set-Up

The study took place in a cork oak (*Quercus suber* L.) woodland situated in southeastern Portugal (38°49′ N, 7°25′ W). The study encompasses an area of approximately 900 ha, where cork oak (*Quercus suber* L.) trees predominate, with some areas mixed with holm oak (*Quercus rotundiolia* Lam). The cork oak trees within the studied area had a density of 160 ± 19 trees ha^−1^, with an average height of 6.6 ± 0.5 m. Additionally, these trees were estimated to be 60 years old. This cork oak woodland area was invaded by the gum rockrose (*Cistus ladanifer* L.) shrub, which forms a dense and mono-specific understory due to its highly competitive and allelopathy characteristics [12,55]. The shrub layer had a density of ca. 11,000 shrubs ha^−1^ (> 90% shrub cover), and an average height of ca. 2–3 m. Shrubs were ca. 15 years old.

The climate is characterized by hot, dry summers and mild, wet winters, a typical Mediterranean climate. The average annual temperature is 16.5 °C and the average annual precipitation is 585 mm [28]. The soil is a shallow (0.4 m deep, on average) haplic leptosol [56], with a high proportion of gravel [15,57].

We conducted a factorial experiment to study the effects of imposed drought and gum rockrose shrub invasion on cork oak trees. We established three replicates of four treatments (12 m × 15 m plots, 12 in total) in a randomized block design: ambient precipitation with a non-invaded cork oak woodland (AQ); drought with a non-invaded cork oak woodland (DQ); ambient precipitation with a gum rockrose invaded cork oak woodland (AQC); and drought with a gum rockrose invaded cork oak woodland (DQC). All the studied plots were colonized by gum rockrose before 2011. In 2011, the gum rockrose shrubs were removed from half of the plots. The drought treatments were imposed by installing nontransparent half-pipe PVC tubes 0.125 m in diameter, mounted 0.40–0.05 m above ground to minimize soil surface interference and ensure water run-off. Rain exclusion started in November 2017, covering 30% of the plot area. In April 2019, rain exclusion was increased to 45% by the addition of half-pipe PVC tubes. A 2.20 m high fence was erected around the blocks to prevent the entry of large animals that inhabit the ecosystem, such as wild boar (*Sus scrofa*), deer (*Cervus elaphus*) and fallow deer (*Dama dama*).

In each plot, three mature cork oak trees and three gum rockrose shrubs (in invaded plots) were selected to conduct measurements (36 cork oak trees and 18 gum rockrose shrubs, in total). Measurements were conducted from November 2019 to September 2020.

### 4.2. Environmental Monitoring

Precipitation, air temperature and relative humidity were measured continuously in three weather stations located in each one of the three blocks and stored every half hour on data loggers, as described in detail in Haberstroh et al. (2021) [15]. Vapor pressure deficit (*VPD*) was calculated from the half-hour averages of air temperature and relative humidity.

### 4.3. Leaf Water Potential

Leaf water potential (*Ψ*) was measured in the 36 cork oak trees and 18 gum rockrose shrubs using a Scholander-type pressure chamber (PMS 1000, PMS Instruments Co., Corvallis, OR, USA) on 6 December 2019, 17 June 2020 and 3 August 2020, hereafter named autumn, spring and summer, respectively. Leaf water potential was measured before sunrise (predawn leaf water potential, *Ψ_pd_*) in two-to-three leaves from mid- to top-canopy height, of each tree and shrub, immediately after excision.

### 4.4. Leaf Gas Exchange

Leaf gas exchange was measured with two cross-calibrated portable photosynthesis systems with a light source and a CO_2_ injector system for controlled CO_2_ concentrations (LI-6400XT, LI-COR Inc, Lincoln, NB, USA). 

Diurnal courses of leaf gas exchange were conducted on the same dates as previously mentioned to cover the periods with water availability and the progression of the drought. The diurnal courses of leaf gas exchange were performed on days with a clear sky, at 10:00, 13:00 and 16:00. These measurements were performed on one-to-two current year fully developed leaves per tree and shrubs from mid- to top-canopy, south exposed, detached branches. The leaves were measured within two minutes after branch cutting. Light intensity (PPFD) was set to 1200 μmol photons m^−2^ s^−1^ (which was found to be saturating for photosynthesis from the A/PPFD curves) and CO_2_ concentration was set to 400 ppm (ambient concentration). The temperature and relative humidity inside the chamber were manually controlled and kept close to ambient conditions. 

From the diurnal courses of leaf gas exchange, the maximal photosynthetic rate (*A_max_*) and the corresponding stomatal conductance (*g_smax_*) were determined. These values were then used to calculate the intrinsic water use efficiency (*iWUE*), which is the ratio of *A_max_* to *g_smax_*.

Net CO_2_ assimilation vs. intercellular CO_2_ concentration (*A/Ci*) response curves were obtained by sequentially increasing the atmospheric CO_2_ concentrations (50, 100, 200, 400, 800, 1200, 1600, 2000 ppm) in the leaf cuvette. PPFD was set to 1200 μmol photon m^−2^ s^−1^. The temperature and relative humidity inside the chamber were manually controlled to match ambient conditions. The biochemical model of Farquhar–von Caemmerer–Berry [48] was fitted to *A/Ci* response curves using the *fitacis* function of the *plantecophys* package in R (R Core Team [58]) to estimate the maximum Rubisco CO_2_ fixation capacity (*V_cmax_*) and maximum electron transport rate (*J_max_*), as described in Duursma et al. (2015) [59]. Mesophyll conductance was assumed to be infinite (i.e., no mesophyll limitation), like in other studies (e.g., [46,60,61]), as we were unable to measure it; hence, the values represent apparent *V_cmax_* and *J_max_*. Leaf temperature was recorded with a thermocouple sensor and the estimated parameters from *A/Ci* response curves were standardized to a leaf temperature of 25 °C, according to Medlyn et al. [62]. The *A/Ci* response curves were measured between 08:00 and 12:00 local time to avoid stomatal closure which might have occurred thereafter. The sampled leaves were in similar conditions to those used for leaf water potential or gas exchange, consisting of two-to-eight replicates of each treatment, per date and per species. Measurements in the trees and in detached branches were compared in all seasons to confirm that the detachment of branches had no effect on the photosynthetic parameters. The periods for measuring response curves were as follows: 27–29 November 2019 (gum rockrose shrubs) and 3–5 and 12 December 2019 (cork oak trees), 19–22 May 2020 (cork oak and gum rockrose), 15–18 September 2020 (cork oak and gum rockrose), hereafter referred to as autumn, spring and summer, respectively.

### 4.5. Leaf Nitrogen Concentration

Leaves from the branches used for A/Ci response curves were collected after the measurement (±30 leaves), kept in a cooling box (4 °C) and transported to the laboratory for leaf area and nitrogen (N) determination. After being scanned and after the leaf area was determined (Winseedle, Regent Instruments Inc., Quebec City, Quebec, Canada), leaves were oven dried for 72 h at 65 °C. Dried leaves were then ground in a ball mill (MM2000, Retsch, Haan, Germany) for N content determination. N content was determined by near-infrared reflectance spectral analysis (NIR), as described in Lobo-do-Vale et al. (2019) [20]. Leaf nitrogen concentration (mg g^−1^) was divided by the area of the leaves of each sample to present the nitrogen data on a leaf area basis, *N_a_* (g m^−2^).

### 4.6. Statistical Analysis

The statistical analysis was performed separately by date and species. To evaluate the combined effects of imposed drought and shrub invasion on cork oak physiological responses and photosynthetic capacity, we used a general linear model (GLM), in which block was considered a random factor and imposed drought, shrub invasion, their interactions and date were considered fixed factors. When a significant drought x shrub invasion interaction or date effect was found, the Tukey HSD test was performed for multiple comparisons of means. To evaluate the effect of imposed drought on gum rockrose physiological responses and photosynthetic capacity, a GLM was also used, with block as a random factor and imposed drought and date as fixed factors. When a significant date effect was found, the Tukey HSD test was performed for multiple comparisons of means. The GLM was repeated to compare cork oak and gum rockrose physiological responses, in which block was considered a random factor and species, date and their interactions were considered fixed factors. Then, to assess the differences between species within the season, pooled data of each species were used to perform Student’s T-test in the variables of interest. Data were log- or squared root-transformed when necessary to meet the assumptions of parametric analyses or the correspondent non-parametric t-test (Mann–Whitney Rank Sum Test). Statistical analyses were carried out with IBM SPSS Statistics 26 (IBM Corp., Armonk, NY, USA). All statistical relationships were considered significant at *p* < 0.05. Data are presented in mean ± SE (standard error of the mean).

## Data Availability

The data presented in this study are available on request to the corresponding author.
